# Development of a High‐Throughput LC–MS/MS Method for Simultaneous Quantification of Four Therapeutic Monoclonal Antibodies in Human Serum: Application in Clinical Therapeutic Drug Monitoring

**DOI:** 10.1002/bmc.70350

**Published:** 2026-02-11

**Authors:** Yuan Yao, Guang‐Yao Huang, Ting‐Ting Wu, Ting‐Fei Tan, Feng‐Mei Hu, Chao Huang, Shan Gao, An‐Ping Guo, Jun‐Ping Wang

**Affiliations:** ^1^ Hefei Institutes of Physical Science Chinese Academy of Sciences, Hefei Cancer Hospital Hefei China; ^2^ Shanghai AB Sciex Analytical Instrument Trading Co. Ltd. Shanghai China; ^3^ Department of Pharmacology, Basic Medical College Anhui Medical University Hefei China; ^4^ Department of Pharmacy The First Affiliated Hospital of University of Science and Technology of China. Anhui Provincial Hospital Hefei China

**Keywords:** bevacizumab, liquid chromatography–tandem mass spectrometry, pertuzumab, rituximab, trastuzumab

## Abstract

Monoclonal antibody (mAb) therapies have revolutionized cancer treatment, significantly improving patient outcomes. However, the pharmacokinetics (PK) and pharmacodynamics (PD) of mAbs exhibit considerable variability due to nonlinear kinetics and individual differences, highlighting the need for therapeutic drug monitoring (TDM). Therefore, this study aimed to develop and validate a reliable LC–MS/MS method for the simultaneous quantification of bevacizumab, trastuzumab, rituximab, and pertuzumab in human serum and evaluate its clinical applicability. Characteristic peptides were identified using Skyline. Serum samples underwent Protein G purification and trypsin digestion. Separation used a C18 column with 0.1% FA and acetonitrile, and detection employed multiple reaction monitoring with cadonilimab as the internal standard. The method demonstrated excellent linearity (1–200 μg/mL), precision (CV < 8.9%), and accuracy (±9.8%). With a runtime of 12 min, the validated method requires only 10 μL of serum per sample and meets international validation standards, supporting the clinical monitoring of these therapies. A robust, cost‐effective, and high‐throughput LC–MS/MS method was successfully developed for the simultaneous quantification of four therapeutic mAbs. The method significantly reduces sample volume and analysis time while maintaining high accuracy and reproducibility, making it well‐suited for routine TDM and broader clinical applications.

**Abbreviations**
CE: collision energy
CXP: collision cell exit potential
DP: declustering potential
ELISA: enzyme‐linked immunosorbent assays
EP: entrance potential
FA: formic acid
ILD: interstitial lung disease
LBAs: ligand‐binding assays
LC–MS/MS: liquid chromatography–tandem mass spectrometry
LLOQ: lower limit of quantification
mAb: monoclonal antibody
mCRC: metastatic colorectal cancer
MRM: multiple reaction monitoring
OS: overall survival
PBS: phosphate‐buffered saline
PD: pharmacodynamics
PFS: progression‐free survival
PK: pharmacokinetics
QAP: quantitative peptides
RE: relative error
RSD: relative standard deviation
TDM: therapeutic drug monitoring

## 1 Introduction

In recent years, monoclonal antibodies (mAbs) have become indispensable therapeutic agents for managing various diseases, particularly cancer and autoimmune disorders. These targeted biologics exhibit remarkable specificity and efficacy, leading to the approval of an increasing number of mAb drugs for clinical use (Fu et al. 2022). Among these, rituximab, trastuzumab, bevacizumab, and pertuzumab have demonstrated significant clinical efficacy in the treatment of conditions such as lymphoma (Jacobsen 2022), breast cancer (Swain et al. 2023), and colorectal cancer (Prager et al. 2023).

Despite their superior clinical efficacy, the pharmacokinetic (PK) profiles of mAbs exhibit substantial interindividual variability (Mir et al. 2020). This variability can result in significant differences in therapeutic efficacy and safety among patients receiving the same dosage. For instance, (Papachristos et al. 2020) reported a positive correlation between bevacizumab concentrations and survival rates in patients with metastatic colorectal cancer (mCRC). Similarly, trastuzumab, a cornerstone therapy for HER2‐positive breast cancer, has been extensively studied for its concentration‐dependent therapeutic effects. Higher exposure to trastuzumab has been shown to improve progression‐free survival (PFS) and overall survival (OS) in HER2‐positive breast cancer patients, although it also increases the risk of adverse events such as interstitial lung disease (ILD) (Yin et al. 2021). Daydé et al. (2009) found that higher serum rituximab concentrations were associated with improved therapeutic outcomes, underscoring the importance of maintaining adequate drug levels, particularly in patients with a high tumor burden. Furthermore, Angelica L. et al., in the NeoSphere trial, demonstrated that higher pertuzumab trough concentrations (C_trough) significantly improved pathological complete response rates in HER2‐positive early breast cancer, highlighting the importance of optimal drug exposure for enhancing therapeutic outcomes (Quartino et al. 2017).

Given this variability, therapeutic drug monitoring (TDM) is essential for optimizing mAb therapy, ensuring that patients receive the most effective dosage tailored to their individual PK responses (Mould 2016). To facilitate TDM studies, the development of efficient and reliable quantitative assays for mAbs is crucial.

Conventional methods, such as enzyme‐linked immunosorbent assays (ELISA), provide high sensitivity and specificity for mAb quantification (Schmitz et al. 2016). However, these methods are often hindered by operational complexity, lengthy protocols, and susceptibility to interference from biological matrix components (Truffot et al. 2021). In contrast, liquid chromatography–tandem mass spectrometry (LC–MS/MS) has emerged as a powerful alternative, providing high sensitivity, accuracy, and the capability for simultaneous detection of multiple compounds (Millet, Khoudour, Bros, et al. 2021), which is particularly advantageous for TDM in patients receiving combination therapies or those requiring monitoring of multiple drugs. Recent studies have successfully developed LC–MS/MS methods for quantifying individual mAbs, including rituximab (Truffot et al. 2021; Millet, Khoudour, Lebert, et al. 2021), trastuzumab (Chiu et al. 2022), and bevacizumab (Zhou et al. 2023), in human serum. General approaches using affinity capture (e.g., Protein G) for IgG‐based mAbs coupled with LC–MS/MS have also been reported (Chiu et al. 2017; Fung et al. 2016), demonstrating the feasibility of multi‐analyte methods. However, a robust, validated LC–MS/MS method specifically designed for the simultaneous quantification of the critical therapeutic quartet—bevacizumab, trastuzumab, rituximab, and pertuzumab—in human serum/plasma is still lacking. Furthermore, existing multi‐analyte Protein G‐LC–MS/MS methods often require long sample preparation times, particularly for enzymatic digestion (e.g., overnight incubation) (Fung et al. 2016), which can limit throughput in a clinical setting.

Therefore, this study aimed to develop and validate a novel, high‐throughput LC–MS/MS method for the simultaneous quantification of these four therapeutically vital mAbs (bevacizumab, trastuzumab, rituximab, and pertuzumab) in human serum. To achieve this, we employed Protein G magnetic beads for efficient capture and purification, significantly optimizing the trypsin digestion step to a rapid 2‐h protocol, substantially enhancing throughput compared to conventional methods. Critically, we introduced cadonilimab, a clinically relevant bispecific antibody (anti‐PD‐1/CTLA‐4 IgG), as a structurally analogous internal standard (IS) to effectively compensate for variability throughout the analytical workflow (capture, digestion, and ionization), offering a cost‐effective alternative to stable isotope‐labeled standards (SILmAbs) (Chen et al. 2024) and differing from the IS strategies used in previous general methods (Fung et al. 2016). The method was rigorously validated according to regulatory guidelines and was successfully applied in quantifying mAb concentrations in clinical samples from cancer patients, revealing substantial interindividual variability even at identical doses, underscoring the clinical need for TDM facilitated by such multiplexed assays.

## 2 Materials and Methods

### 2.1 Reagents and Materials

Bevacizumab, trastuzumab, and pertuzumab were obtained from Roche Applied Science (USA), while rituximab was sourced from Shanghai Fuhong Hanlin Biopharmaceutical Co. Ltd. (Shanghai, China). Pancreatin was purchased from Solarbio (USA). Protein G Mag Sepharose beads were supplied by GE Healthcare (USA). LC–MS/MS‐grade methanol and acetonitrile were acquired from Merck (Germany). HyClone phosphate‐buffered saline (PBS) was obtained from Cytiva (USA). Bovine serum albumin, ammonium bicarbonate, and 99% formic acid (FA) solution were procured from Sigma (USA).

### 2.2 LC–MS/MS System

The analysis was conducted using an AB Sciex 4500 MD system, operated with Analyst 1.6.2 software (AB Sciex, Framingham, MA, USA). Chromatographic separation was performed on a PEPTIDE XB‐C18 column (100 × 2.1 mm, 1.7 μm particle size; Phenomenex, Torrance, USA). The system operated in positive electrospray ionization (ESI) mode under the following conditions: ESI voltage, 5500 V; ion source temperature, 600°C; gas 1 (heater gas), 65 psi; gas 2 (curtain gas), 30 psi; and nebulizer gas (GS1), 60 psi. Quantification was performed in multiple reaction monitoring (MRM) mode. The mobile phase consisted of solvent A (0.1% FA in water) and solvent B (0.1% FA in acetonitrile), delivered at a flow rate of 0.3 mL/min. The gradient elution program was as follows: 0–1 min, 10% B; 1–7.5 min, 10%–30% B; 7.5–8 min, 30%–90% B; 8–10 min, 90% B, followed by a 2‐min re‐equilibration at 10% B. The autosampler was maintained at 4°C during the analysis, and the column compartment temperature was set to 40°C. The sample injection volume was 20 μL. Peptide candidates for quantification were selected based on the internal optimization of trypsin‐digested peptides. Table 1 provides details of the protein‐derived peptides used for quantification, their respective MRM transitions, and specific MRM settings.

**TABLE 1:** Mass spectrometry parameters and proteotypic peptides: MRM transitions and voltage settings, including declustering potential (DP), entrance potential (EP), collision energy (CE), and collision cell exit potential (CXP) for each analyte.

mAb drug	mAb mass (Da)	Proteotypic peptide	Q1 m/z	Q3 m/z	CE (eV)
Bevacizumab	149,000	FTSLDTSK	523.264	797.404	24.6
			523.264	650.336	24.6
Trastuzumab	145,531.5	FTISADTSK	485.248	721.373	22.8
			485.248	608.299	25.8
Pertuzumab	148,000	GLEWVADVNPNSGGSIYNQR	1088.525	1306.613	49.3
			1088.525	1192.571	52.3
Rituximab	143,859.7	FSGSGSGTSYSLTISR	803.889	1027.542	44.4
			803.889	926.494	44.4
Cadonilimab	/	SLIGGTNNK	452.248	590.299	24.2
			452.248	533.268	24.1

### 2.3 Computer‐Simulated Trypsin Digestion

Trypsin digestion was simulated using Skyline software (version 3.5, MacCoss Lab, University of Washington, WA, USA). The human plasma background proteome was excluded using the PeptideAtlas human plasma background protein database (HumanPlasma_2012 2008, http://www.peptideatlas.org/speclib/). Specific peptides were selected after filtration. Fragmentation and collision energies for each peptide were optimized in mass spectrometry to achieve maximum signal intensity. Detailed information for each selected peptide is provided in Table 1.

### 2.4 Calibration Curve and Quality Control

Bevacizumab (25 mg/mL), trastuzumab (22 mg/mL), pertuzumab (12.5 mg/mL), and rituximab (10 mg/mL) were diluted with blank plasma to 200 μg/mL to prepare the working solution for the standard curve. This solution was further diluted with blank plasma to produce calibration standards at concentrations of 1, 2, 4, 10, 50, 100, and 200 μg/mL. Quality control (QC) samples were similarly prepared by diluting the working solution to 160 μg/mL (high), 10 μg/mL (medium), and 3 μg/mL (low), with a lower limit of quantification (LLOQ) of 1 μg/mL.

### 2.5 IS Preparation

Cadonilimab (12.5 mg/mL) was diluted with water to prepare an IS solution at a concentration of 50 μg/mL.

### 2.6 Sample Preparation

**FIGURE 1:**
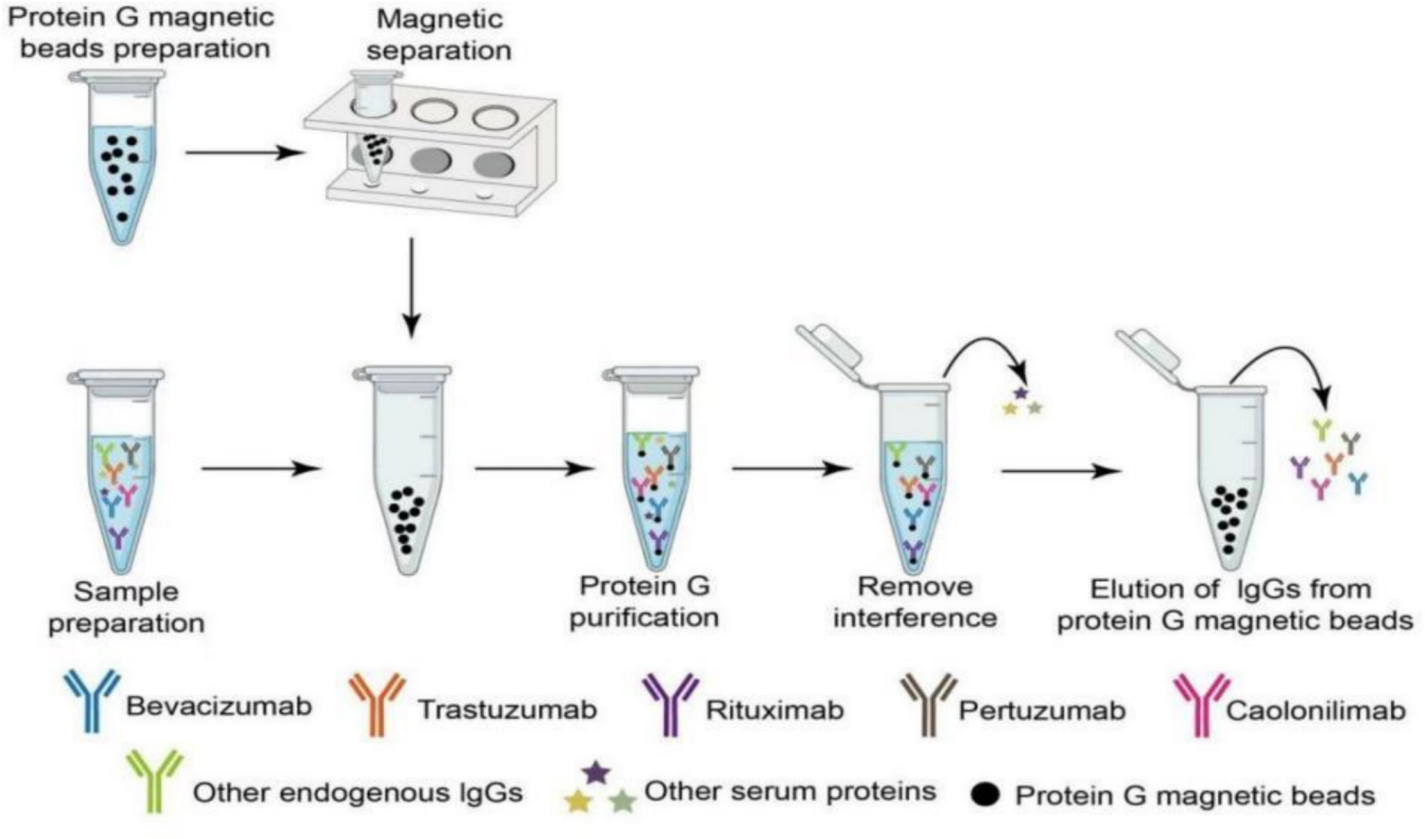
Schematic representation of Protein G capturing of four monoclonal antibody drugs (bevacizumab, trastuzumab, pertuzumab, and rituximab) and the internal standard (cadonilimab) from human plasma.

Protein G beads were utilized to capture bevacizumab, trastuzumab, pertuzumab, and rituximab from human plasma. A schematic diagram of the capture process for these four monoclonal antibodies and the IS, cadonilimab, is presented in Figure 1. Forty microliters of Protein G bead solution (prewashed twice with 500‐μL PBS) were mixed with 20 μL of the sample, 10 μL of the IS solution, and PBS containing 0.05% Tween 20. The mixture was incubated at room temperature with shaking at 900 rpm for 1 h. After incubation, the supernatant was discarded, and the beads were washed twice with 500‐μL PBS containing 0.1% BSA and 500‐μL deionized water to remove unbound proteins. Elution of the monoclonal antibodies was achieved by adding 100 μL of 0.25% FA, and 90 μL of the eluent was transferred to a new microtube. Neutralization was performed by adding 17 μL of ammonium bicarbonate solution, followed by heating at 95°C with shaking at 900 rpm for 30 min. Subsequently, 15 μL of 0.5‐mg/mL trypsin was added, and the sample was digested at 37°C with shaking at 900 rpm for 2 h. Digestion was halted by adding 23 μL of a 10% FA‐acetonitrile‐water solution. The mixture was vortexed and centrifuged at 12,000 rpm for 5 min, and the supernatant was collected for LC–MS/MS analysis.

### 2.7 Method Validation

The analytical method was validated according to FDA guidelines (U.S. Food and Drug Administration 2018) focusing on specificity, linearity, LLOQ, accuracy, precision, carryover, and matrix effects. Validation was conducted exclusively on quantitative peptides (QAP).

#### 2.7.1 Specificity

Specificity were assessed using six blank human plasma samples to evaluate potential interferences from the extraction process and analytical conditions on analyte peak areas.

#### 2.7.2 Linearity and LLOQ

Calibration points ranging from 1 to 200 μg/mL were used to construct the standard curve. The LLOQ, defined as the lowest concentration on the calibration curve, was evaluated over three consecutive days. Daily calibration curves were analyzed using least squares linear regression, with the regression coefficient (*R* ^2^) consistently exceeding 0.99. Each concentration within the linear range met predetermined precision and accuracy limits (typically ±15% and ±20% for the LLOQ).

#### 2.7.3 Precision and Accuracy

The method's accuracy and precision were evaluated using low, medium, and high concentration QC samples. Repeat analyses (*n* = 5) were performed both within the same day (within‐day) and over three separate validation days (between‐day). Accuracy and precision at each QC level were reported as relative error (RE) and relative standard deviation (RSD), respectively. Both RE and RSD were required to fall within ±15% of the nominal concentration, with a tolerance of ±20% for the LLOQ.

#### 2.7.4 Carryover Effects

Carryover was assessed by injecting three blank samples following ULOQ samples. Residual analyte responses were required to be ≤ 20% of the analyte signal and ≤ 5% for the IS.

#### 2.7.5 Stability

The stability of the four monoclonal antibodies in plasma was assessed by analyzing QC samples under various storage and handling conditions. Freeze–thaw stability was tested after three freeze–thaw cycles (from −80°C to room temperature). Long‐term stability was evaluated by storing samples at −20°C for 30 days. Stability of prepared samples was determined by analyzing extracted QC samples stored in an autosampler at 4°C for 24 h and comparing concentrations to those of freshly prepared QC samples. Stability was expressed as the percentage deviation from the initial concentrations.

#### 2.7.6 Matrix Effect and Extraction Recovery

Matrix effects were evaluated by spiking three concentrations of monoclonal antibodies into six blank plasma and reagent blank samples. The matrix effect was expressed as the ratio of the average peak area of analytes extracted from biological samples to that of analytes in equivalent concentration samples prepared from blank reagents. Extraction recovery was assessed by spiking the four monoclonal antibodies at three concentrations (3, 10, and 160 μg/mL) into plasma samples, followed by the addition of bevacizumab to blank plasma after Protein G capture. Recovery was calculated by dividing the peak area of pre‐spiked samples by that of post‐spiked samples and multiplying by 100%.

### 2.8 Clinical Sample Application

Plasma samples were obtained from patients at our hospital who had received at least 1 cycle of bevacizumab, trastuzumab, rituximab, or pertuzumab. Blood samples were drawn within 30 min before drug administration. The collected blood was centrifuged at 3000 rpm for 10 min at 4°C to separate the supernatant, which was transferred to 1.5‐mL EP tubes and stored at −80°C until analysis.

## 3 Results and Discussion

### 3.1 Optimization of Chromatographic and Mass Conditions

MRM conditions were optimized for the simultaneous quantification of four mAbs in plasma. To optimize mass spectrometry (MS) conditions, both positive and negative ion modes were evaluated. Fullscan MS analysis indicated that positive ion mode yielded higher ionization efficiency for the four mAbs and the IS. Collision energy parameters were fine‐tuned to maximize the relative abundance of precursor and product ions.

In LC–MS/MS analysis, the use of an IS was essential for ensuring accuracy. We introduced cadonilimab, a bispecific antibody, as a structurally analogous IS. Protein G capture combined with this IS effectively corrected variations from the protein capture phase to the trypsin digestion phase, ensuring reliable results. The selected IS exhibited excellent performance in terms of linearity and quantification accuracy.

### 3.2 Optimization of Sample Preparation

Sample preparation is critical for accurate and reliable LC–MS/MS analysis. Initial attempts showed significant interference peaks for all surrogate peptides, which could not be resolved chromatographically, hindering quantification. To improve selectivity and achieve lower limits of quantification, rituximab, trastuzumab, bevacizumab, and pertuzumab were enriched from human serum samples using magnetic beads. The enriched mAbs underwent protein denaturation, reduction, alkylation, and enzymatic digestion, with optimal conditions for temperature and duration established for each step. LC–MS/MS was then utilized to accurately quantify the mAbs in human serum, demonstrating the effectiveness of the optimized sample preparation process.

### 3.3 Method Validation

#### 3.3.1 Specificity

**FIGURE 2:**
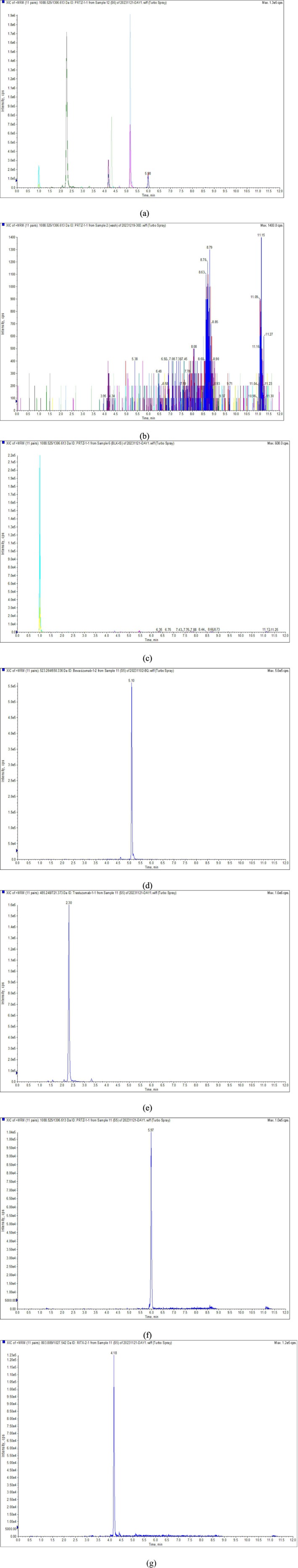
The total ion chromatogram (a), blank plasma (b), and blank plasma with internal standard (c), and the selected proteotypic peptides of bevacizumab (d), trastuzumab (e), pertuzumab (f), and rituximab (g).

The specificity of the method was evaluated using six blank plasma samples, which confirmed the absence of endogenous or external interferences at the retention times of the analytes and the IS. The retention times were as follows: rituximab at 4.18 min, trastuzumab at 2.30 min, bevacizumab at 5.20 min, pertuzumab at 5.97min, and the IS at 1.03 min. Representative chromatograms are shown in Figure 2, including the total ion chromatogram (Panel a), a blank sample (Panel b), and the blank sample with IS (Panel c), and individual chromatograms for bevacizumab (Panel d), trastuzumab (Panel e), pertuzumab (Panel f), and rituximab (Panel g).

#### 3.3.2 Linearity and LLOQ

The linear ranges for the analytes were determined as follows: pertuzumab (y = 0.0362x − 0.011), trastuzumab (y = 0.623x + 0.129), bevacizumab (y = 0.51x − 0.0883), and rituximab (y = 0.0417x − 0.0103), where y represents the analyte‐to‐IS peak area ratio, and x represents the analyte concentration in plasma. The correlation coefficients (*r*) for all calibration curves exceeded 0.995, demonstrating excellent linearity across the specified concentration ranges. LLOQ corresponded to the lowest concentrations on the calibration curves and exhibited good within‐day and between‐day accuracy and precision (Table 2).

**TABLE 2:** Precision and accuracy for the four monoclonal antibodies in plasma.

mAb drug		Within‐day (*n* = 5)			Between‐day (*n* = 5)		
	Theoretical concentration	Measured concentration	Precision	Accuracy	Measured concentration	Precision	Accuracy
	(μg/mL)	(μg/mL)	(RSD, %)	(RE, %)	(μg/mL)	(RSD, %)	(RE, %)
Pertuzumab	3	2.80 ± 0.1	4.2	−7.5	3.0 ± 0.2	7.6	0.3
	10	10.2 ± 0.3	2.5	2.1	9.9 ± 0.6	5.9	−1.4
	160	175.6 ± 1.5	0.8	9.8	158.6 ± 14.2	8.9	−0.9
Trastuzumab	3	2.7 ± 0.1	3.0	−9.8	3.1 ± 0.3	9.0	1.9
	10	10.2 ± 0.3	2.7	2.4	10.0 ± 0.5	5.2	−0.3
	160	161.2 ± 7.5	4.6	0.8	156.6 ± 9.8	6.3	−2.1
Bevacizumab	3	3.0 ± 0.3	9.4	0.5	3.1 ± 0.2	6.1	1.8
	10	10.1 ± 0.3	2.5	0.7	9.7 ± 0.7	7.6	−3.0
	160	161.4 ± 3.3	2.0	0.9	158.9 ± 8.5	5.4	−0.7
Rituximab	3	2.8 ± 0.1	3.2	−5.6	3.0 ± 0.2	6.2	−0.1
	10	10.8 ± 0.1	1.3	8.0	10.3 ± 0.6	5.4	2.8
	160	163.2 ± 8.8	5.4	2.0	153.1 ± 10.4	6.8	−4.3

*Note:* All RSD values were within acceptable limits (±15%), demonstrating good precision of the method.

#### 3.3.3 Precision and Accuracy

Table 2 summarizes the within‐day and between‐day precision and accuracy of the analytes in QC samples at three concentration levels. Within‐day precision ranged from 0.8% to 9.4%, while between‐day precision ranged from 5.2% to 8.9%. Within‐day accuracy varied from −9.8% to 9.8%, and between‐day accuracy ranged from −4.3% to 2.8%. These results demonstrate that the measured values meet established criteria, confirming that the method is accurate, reliable, and highly reproducible.

#### 3.3.4 Matrix Effect and Extraction Recovery

The matrix effect was evaluated at three concentration levels, with results ranging from 90.05% to 109.79%. Extraction recovery using the Protein G capture method was tested at the same concentration levels, yielding recovery rates between 88.57% and 106.68%.

#### 3.3.5 Stability

The stability of the four monoclonal antibodies in plasma was assessed using QC samples under various storage and handling conditions. As shown in Table 3, the analytes remained stable after three freeze–thaw cycles, 24‐h storage in a 4°C autosampler, and 30‐day storage at −20°C. These findings confirm the robustness of plasma samples for extended analysis.

**TABLE 3:** Stability of four monoclonal antibodies in plasma.

mAb drug	Theoretical concentration	Autosampler | 24 h	Freeze–thaw cycles | 3 times	−20°C | 30 days
	(μg/mL)	RSD (%)	RSD (%)	RSD (%)
Pertuzumab	3	6.6	3.9	4.8
	160	2.4	2.5	2.1
Trastuzumab	3	2.2	13.6	5.7
	160	1.3	5.1	1.2
Bevacizumab	3	6.0	3.76	4.5
	160	3.4	6.3	2.7
Rituximab	3	5.4	3.9	1.1
	160	2.4	3.3	4.4

### 3.4 Clinical Sample Analysis

Patient information, including gender, age, and mAb dosage, was collected. This study was conducted at Hefei Cancer Hospital, Chinese Academy of Sciences, from March 1, 2023, to October 20, 2024, and received approval from the Institutional Ethics Committee (Approval No: PJ‐XJS2024‐021).

The established LC–MS/MS method was applied to analyze five samples of bevacizumab, seven samples each of trastuzumab, rituximab, and pertuzumab. The results are summarized in Table 4. The method accurately quantified the plasma concentrations of the four mAbs, with calibration curves and QC samples meeting all required standards. Among patients receiving identical doses of rituximab (600 mg), plasma concentrations varied widely (15.5–223 μg/mL; CV = 78%), suggesting large interindividual PK variability possibly related to tumor burden or clearance rate differences. Similarly, bevacizumab levels ranged from 28.4 to 67.8 μg/mL despite uniform dosing, consistent with previous observations in mCRC patients (Papachristos et al. 2020).

**TABLE 4:** Measured concentrations of four monoclonal antibodies (26 clinical samples).

mAb drug	Patient no.	Gender	Age	Dose (mg)	Measured concentration
					(μg/mL)
Bevacizumab	1	F	57	400	36.4
	2	M	47	450	67.8
	3	F	34	400	28.4
	4	M	27	400	56.6
	5	F	69	400	29.4
Trastuzumab	1	F	59	450	40
	2	F	55	380	52.6
	3	F	81	300	64.3
	4	F	55	300	41.2
	5	F	68	420	32.6
	6	F	33	426	33
	7	F	55	390	60.3
Pertuzumab	1	F	59	420	32.5
	2	F	55	420	77.4
	3	F	81	420	129
	4	F	55	420	105
	5	F	68	420	32.3
	6	F	33	420	27.6
	7	F	55	420	78.6
Rituximab	1	F	51	600	50.8
	2	F	49	600	25.2
	3	M	55	600	223
	4	M	57	600	75
	5	F	67	600	39.4
	6	F	64	600	77.3
	7	F	59	600	15.5

## 4 Discussion

TDM is increasingly recognized as a crucial tool for optimizing the efficacy and safety of mAb therapies, given their significant interindividual pharmacokinetic variability (Mir et al. 2020; Mould 2016). While ligand‐binding assays (LBAs) such as ELISA are widely used for mAb quantification due to their throughput and accessibility (Schmitz et al. 2016; Vande Casteele et al. 2012), they are hampered by several limitations (Table 5). These include susceptibility to matrix interferences, limited dynamic range, and typically a single‐analyte focus, which complicates the monitoring of combination regimens (Truffot et al. 2021; Vande Casteele et al. 2012). In contrast, LC–MS/MS offers superior specificity, the ability to differentiate between closely related molecules (Millet, Khoudour, Bros, et al. 2021; Hallin et al. 2024), and—most importantly—the capability for multiplexing. This multiplexing advantage is exemplified when comparing our approach to recently reported UPLC–MS/MS methods for simultaneous mAb quantification (Buitelaar et al. 2024; Liu et al. 2022): While offering comparable throughput and sensitivity, our method enables the simultaneous quantification of four analytes in a single run and employs a structurally analogous IS for enhanced accuracy. Generalizable LC–MS/MS platforms utilizing affinity capture (e.g., Protein G) for IgG‐based mAbs represent a significant advancement towards addressing the need for efficient TDM tools (Chiu et al. 2017; Fung et al. 2016; Chiu et al. 2018). However, a dedicated, validated method for the simultaneous quantification of the critical therapeutic quartet—bevacizumab, trastuzumab, rituximab, and pertuzumab—was lacking. Furthermore, existing methods often require protracted sample preparation, notably lengthy enzymatic digestion (e.g., overnight) (Fung et al. 2016), limiting their utility in high‐throughput clinical environments.

**TABLE 5:** Comparison of key characteristics between ELISA and the developed LC–MS/MS method for therapeutic mAb quantification.

Parameter	ELISA methods	LC–MS/MS method	References
Multiplexing capability	Typically single‐analyte; multiplex ELISAs complex	Simultaneous quantification of four mAbs in a single run	This study; Aydin et al. 2025
Sensitivity	Least sensitive	Highly sensitive	This study; Aydin et al. 2025
Total analysis time	4–8 h (incubation steps)	12 min runtime + 2 h digestion	This study; Aydin et al. 2025
Specificity	Susceptible to matrix interference	High specificity via proteotypic peptides + Protein G purification	This study; Puszkiel et al. 2017
Internal standard (IS)	Rarely used	Structurally analogous IS (cadonilimab) corrects variability	This study; Aydin et al. 2025

This study successfully addresses this gap through the development and validation of a novel, high‐throughput LC–MS/MS method specifically designed for the simultaneous quantification of these four mAbs in human serum. Our method introduces several key innovations that enhance its practicality, efficiency, and robustness for clinical TDM:

### 4.1 Rapid High‐Throughput Workflow

A major advancement is the optimization of the trypsin digestion step to a rapid 2‐h protocol. This is a substantial reduction compared to the often lengthy (e.g., 14–18 h or overnight) digestions required in previous Protein G‐based LC–MS/MS methods (Fung et al. 2016). This optimization significantly enhances sample throughput without compromising the rigorous accuracy and precision demonstrated during validation (Tables 2 and 3).

### 4.2 Novel and Cost‐Effective IS Strategy

We employed cadonilimab, a clinically available bispecific IgG antibody (anti‐PD‐1/CTLA‐4), as a structurally analogous IS. Unlike expensive stable isotope‐labeled standards (SILmAbs) (Chen et al. 2024), cadonilimab provides a readily available and cost‐effective alternative. Its IgG structure allows it to effectively track and correct for variability throughout the analytical workflow—Protein G capture, enzymatic digestion, and ionization—similar to the principle of using another therapeutic mAb as an IS (Chiu et al. 2017; Fung et al. 2016). This strategy differs from previous approaches and significantly enhances the method's reliability.

### 4.3 Enhanced Efficiency and Clinical Utility

The synergy of Protein G capture, rapid digestion, and effective IS correction resulted in an exceptionally efficient method. It requires only 10 μL of serum and achieves a total chromatographic runtime of just 12 min. The method demonstrates excellent performance across a wide linear range (1–200 μg/mL) covering clinically relevant concentrations, with precision (CV < 8.9%) and accuracy (±9.8%) meeting international guidelines (U.S. Food and Drug Administration 2018). These attributes make it highly suitable for routine use in clinical TDM laboratories processing large volumes of samples.

The rigorous validation confirms the method's accuracy, precision, and reproducibility. Its successful application to clinical samples from cancer patients effectively quantified the target mAbs and, crucially, revealed substantial interindividual variability in plasma concentrations (Table 4). Notably, we observed up to a twofold difference in concentrations (e.g., bevacizumab: 28.4–67.8 μg/mL; pertuzumab: 27.6–129 μg/mL; rituximab: 15.5–223 μg/mL) among patients receiving identical doses. This finding powerfully underscores the critical need for TDM to optimize dosing strategies for these potent therapeutics and highlights the value of our multiplexed assay in efficiently capturing this variability to guide personalized treatment.

It is also important to consider the applicability of this method to biosimilar versions of the monitored mAbs. Biosimilars are highly similar but not identical to their reference products, and minor structural variations could theoretically influence tryptic digestion efficiency or peptide yield. The proteotypic peptides selected in this method reside in conserved regions of the antibodies, suggesting that the assay should be applicable to common biosimilars. Nevertheless, for rigorous TDM of a specific biosimilar, validation using calibration standards and quality controls prepared from that biosimilar is recommended to ensure accurate quantification.

While the current method focuses on four specific IgG1 mAbs, the core strategy—Protein G capture coupled with a structurally analogous therapeutic mAb IS (like cadonilimab) and an optimized rapid digestion protocol—is inherently adaptable. This platform can be extended to the quantification of other IgG‐based therapeutic antibodies, enhancing its utility as a generalizable tool for bioanalysis.

In conclusion, the advancements presented here establish our LC–MS/MS method as a cost‐effective, time‐efficient, and reliable solution for the high‐throughput TDM of bevacizumab, trastuzumab, rituximab, and pertuzumab. By providing a practical tool for simultaneous quantification, this method paves the way for broader adoption in clinical settings and facilitates further research into exposure–response relationships, ultimately contributing to more personalized and effective cancer treatment.

## 5 Conclusion

This study presents a validated high‐throughput LC–MS/MS method for the simultaneous TDM of four key mAbs. A pivotal technical achievement was the optimization of chromatographic and mass spectrometric conditions—most notably the carefully designed 12‐min separation and MRM detection protocol—which was instrumental in achieving the high specificity, sensitivity, and throughput that define this assay. Building upon this robust analytical core, the method demonstrated excellent precision, accuracy, linearity, and sensitivity, which were rigorously validated. Its clinical application revealed significant interindividual variability in the plasma concentrations of the four mAbs, with differences of up to twofold even among patients receiving identical doses.

Despite its advantages, this study has several limitations. First, the sample size was modest (*n* = 26), and only pre‐dose plasma concentrations were analyzed. Second, the current method was not directly compared with commercial ELISA kits, which could further verify accuracy and clinical correlation. Third, the method was validated for four IgG1‐type antibodies; its applicability to other subclasses (e.g., IgG2 or bispecific formats) warrants further investigation.

This robust method has the potential to be extended for monitoring drug exposure levels of various mAb therapies. By providing accurate and reliable quantification, it enhances the understanding of variability in therapeutic responses, advancing precision medicine in mAb‐based treatments. Future studies will focus on expanding this high‐throughput platform to additional therapeutic antibodies and exploring its integration with pharmacokinetic modeling and AI‐based exposure–response analysis to further advance precision dosing in oncology.

## Author Contributions

**Yuan Yao:** methodology, formal analysis, visualization, writing – original draft, writing – review and editing. **Ting‐Ting Wu:** writing – review and editing. **Feng‐Mei Hu:** methodology, formal analysis. **Chao Huang:** methodology, formal analysis. **Jun‐Ping Wang:** writing – review and editing. **Guang‐Yao Huang:** writing – review and editing. **Shan Gao:** conceptualization, resources, methodology, writing – original draft, writing – review and editing, project administration. **An‐Ping Guo:** conceptualization, resources, methodology, writing – original draft, writing – review and editing, project administration.

## Conflicts of Interest

The authors declare no conflicts of interest.

## Data Availability

Data will be made available on request.
